# Comprehensive Analysis of Immune-Related Mitochondrial Genes in Ischemic Stroke Through Integrated Bioinformatics and Validation

**DOI:** 10.3390/biomedicines14020375

**Published:** 2026-02-05

**Authors:** Chenchen Li, Runfa You, Xianghua Meng, Haowen Long, Chao Zheng, Zijie Zhan

**Affiliations:** 1 Department of Neurology, The Second Xiangya Hospital, Central South University, Changsha 410011, China; lichenchen@csu.edu.cn; 2 Clinical Medical Research Center for Stroke Prevention and Treatment of Hunan Province, Department of Neurology, The Second Xiangya Hospital, Central South University, Changsha 410011, China; 3 Department of Radiology, Changde Hospital, Xiangya School of Medicine, Central South University (The First People’s Hospital of Changde City), Changde 415003, China; youyouyourf@163.com (R.Y.); liuboemailforjob@163.com (X.M.); cdyylonghaowen@163.com (H.L.)

**Keywords:** ischemic stroke, mitochondria, immune infiltration, machine learning, biomarkers

## Abstract

**Background:** Ischemic stroke (IS) is a major cause of disability and mortality worldwide, with mitochondrial dysfunction playing a critical role in its pathogenesis. This study aimed to identify immune-related mitochondrial biomarkers associated with IS and evaluate their diagnostic potential. **Methods:** IS-related gene expression datasets were obtained from the GEO database. Differentially expressed genes (DEGs) were identified from the GSE58294 dataset, followed by functional enrichment analysis, immune infiltration assessment, and weighted gene co-expression network analysis (WGCNA). Immune-related mitochondrial genes were screened using the MITOCARTA 3.0 database. Four machine learning algorithms—random forest (RF), support vector machine (SVM), generalized linear model (GLM), and extreme gradient boosting (XGB)—were applied to identify hub genes. External validation was performed using the GSE16561 dataset, and RT-qPCR confirmed key gene expression. Functional enrichment and single-cell RNA sequencing analyses explored biological pathways and cellular localization. **Results:** Five key genes (*ECHDC3*, *EPHX2*, *SPTLC2*, *MSRB2*, and *TK2*) were identified, among which *ECHDC3*, *EPHX2*, and *SPTLC2* showed strong diagnostic potential (AUC > 0.7). These genes were significantly enriched in apoptosis, JAK-STAT, MAPK, and VEGF signaling pathways and were closely associated with neutrophil infiltration. Single-cell analysis revealed increased immune cell populations and distinct expression patterns of key genes in the ischemic mouse brain. **Conclusions:** This study identifies novel immune-related mitochondrial biomarkers for IS, providing insights into its pathogenesis and offering potential targets for early diagnosis and therapeutic intervention.

## 1. Introduction

Stroke is the second leading cause of death globally, with high mortality and disability rates, posing a significant burden on society and the economy [1]. Ischemic stroke (IS), caused by acute arterial occlusion, accounts for 60–70% of all strokes [2]. Although endovascular reperfusion therapy can improve outcomes in some patients, treatment options for IS remain limited, and the prognosis is often poor due to a narrow therapeutic window, potential bleeding risks, and reperfusion injury after vessel recanalization [3,4]. Therefore, it is necessary to explore novel therapeutic strategies to actively mitigate neuronal damage.

Ischemic injury involves various pathological mechanisms, including excitotoxicity, neuroinflammation, oxidative stress, and different forms of cell death [5,6,7,8]. Among these, mitochondrial dysfunction is considered a pivotal factor. As the primary energy hub of the cell, mitochondria are essential for maintaining normal physiological functions, not only generating ATP but also regulating calcium homeostasis, reactive oxygen species (ROS) production, and apoptosis [9,10]. Following IS, mitochondrial structure and function are compromised, leading to energy metabolism disruption, oxidative stress, and cell death, ultimately exacerbating brain tissue damage [11,12]. Thus, investigating the role of mitochondrial-related genes in IS is crucial for unraveling its pathological mechanisms and identifying novel therapeutic targets.

In recent years, the rapid advancement of high-throughput sequencing technologies has empowered bioinformatics analysis as a robust tool for identifying disease-related genes and deciphering molecular mechanisms. By integrating multi-omics data, such as gene expression profiles and protein–protein interaction networks, key disease-associated genes can be systematically screened, and molecular regulatory networks can be constructed to uncover potential pathological mechanisms and therapeutic targets. This study aims to employ bioinformatics methods to identify mitochondrial-related genes associated with IS and investigate their expression changes and potential functions. We will first download gene expression profiles of IS patients’ blood from the Gene Expression Omnibus (GEO) database and perform differential expression analysis using R to identify differentially expressed mitochondrial-related genes. Subsequently, Gene Ontology (GO) and Kyoto Encyclopedia of Genes and Genomes (KEGG) pathway analyses will be conducted for functional annotation and pathway enrichment, followed by the construction of protein–protein interaction networks to pinpoint key mitochondrial-related genes. Finally, reverse transcription quantitative real-time PCR (RT-qPCR) will be utilized to experimentally validate these key genes and explore their potential roles in IS. The findings of this study will help elucidate the functions of mitochondrial-related genes in IS, providing new insights and theoretical foundations for early diagnosis, prognosis evaluation, and the identification of therapeutic targets.

## 2. Materials and Methods

### 2.1. Dataset Collection

**Figure 1:**
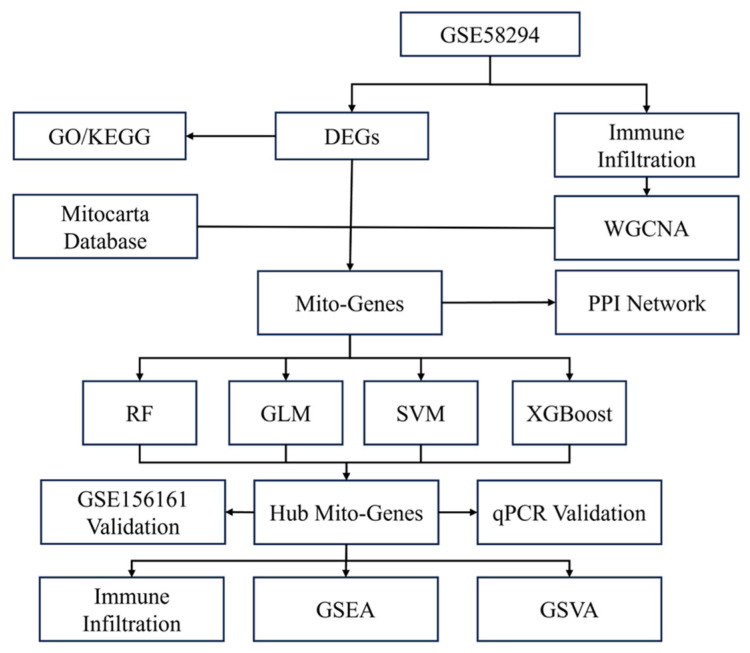
Flow chart and design of the study. DEGs: differentially expressed genes. WGCNA: Weighted Gene Co-expression Network Analysis; PPI: protein–protein interaction. Mito: mitochondrial. RF: random forest. GLM: generated linear model. SVM: support vector machine. XGBoost: extreme gradient boosting. GSEA: gene set enrichment analysis. GSVA: gene set variation analysis.

Gene expression matrices and clinical data from two gene expression array datasets, GSE58294 and GSE16561, were downloaded from the National Center for Biotechnology Information (NCBI) Gene Expression Omnibus (GEO, http://www.ncbi.nlm.nih.gov/geo/, accessed on 5 September 2025) database [13]. The GSE58294 dataset is based on the GPL570 platform (Affymetrix Human Genome U133 Plus 2.0 Array, Affymetrix Inc, Santa Clara, CA, USA) and contains gene expression profiles from peripheral blood samples of 23 control subjects and 69 patients with cardioembolic stroke. Data were collected at three time points after stroke occurrence, 3 h, 5 h, and 24 h, with 23 replicated samples per time point. The GSE16561 dataset is based on the GPL6883 platform (Illumina HumanRef-8 v3.0 expression beadchip, Illumina Inc., San Diego, CA, USA) and includes peripheral whole-blood samples from 39 patients with MRI-confirmed acute ischemic stroke and 24 neurologically healthy controls. GSE58294 was used as the exploratory dataset, while GSE16561 served as the external validation dataset. Figure 1 illustrates the overall flowchart of this study.

### 2.2. Data Preprocessing

The downloaded gene expression matrices were initially assessed for missing values. In cases where missing data were present, multiple imputation was performed using the “MICE” package (version 3.16.0) in the R software (version 4.3.2) with the predictive mean matching method [14]. Subsequently, probe IDs from the microarray platforms were annotated to official gene symbols. During this step, only probes corresponding to unique gene symbols were retained; for probes mapping to the same gene symbol, the expression value of the first probe was retained to represent the gene’s expression level. To ensure comparability across samples, gene expression values were standardized using the normalizeBetweenArrays function from the “limma” package (version 3.62.1). This normalization procedure adjusted for technical variations between arrays, thereby enhancing the reliability of downstream analyses.

### 2.3. Gene Expression Analysis

Differentially expressed genes (DEGs) were identified from the preprocessed GSE58294 dataset using the “limma” package in R [15]. Samples collected at the three post-stroke time points (3 h, 5 h, and 24 h) were merged into a single stroke group. Gene expression levels between this combined stroke group and the control group were compared. DEGs were defined as genes meeting the following criteria: a Benjamini–Hochberg (BH)-adjusted *p*-value < 0.05 and an absolute log2 fold change (|log2FC|) > 0.5.

### 2.4. GO Functional and KEGG Pathway Enrichment Analysis

Gene function of DEGs was enriched using the “clusterProfiler” package (version 4.14.3) [16], with the results visualized using the “enrichplot” package (version 1.26.2). Functional enrichment analysis included Gene Ontology (GO) terms and Kyoto Encyclopedia of Genes and Genomes (KEGG) pathways. GO analysis covered three domains: biological processes (BPs), cellular components (CCs), and molecular functions (MFs). Terms with a Benjamini–Hochberg (BH)-adjusted *p*-value < 0.05 were considered statistically significant.

### 2.5. Immune Infiltration Analysis

The relative proportions of 22 immune cell types in each sample were estimated using the CIBERSORT algorithm (https://cibersort.stanford.edu/, accessed on 5 September 2025), a well-established computational method for deconvoluting immune cell compositions based on gene expression profiles. The CIBERSORT results were visualized through a correlation heatmap generated with the “corrplot” package (version 0.95) to illustrate the relationships between different immune cell populations. Associations between the identified hub genes and immune cell infiltration were analyzed and visualized using lollipop plots created with the “ggplot2” (version 3.5.1) and “ggpubr” (version 0.6.0) packages. Furthermore, the immune infiltration scores derived from CIBERSORT were incorporated into Weighted Gene Co-expression Network Analysis (WGCNA) to identify immune-related gene modules associated with specific immune cell infiltration patterns.

### 2.6. WGCNA

In this study, the step-by-step WGCNA algorithm was employed to construct an unsupervised co-expression network to identify gene modules associated with IS and immune infiltration. The top 25% of genes with the greatest variance in the GSE58294 dataset were selected, and data quality was controlled using the “goodSamplesGenes” function from the WGCNA package (version 1.73) [17]. Then, these gene expression matrices were transformed into a scale-free network using a soft threshold of 0.85, a height cutoff of 0.2, and a minimum module size of 100 genes. Subsequently, the Spearman correlation analysis was used to identify gene modules with the strongest correlation to clinical features and immune infiltration of IS. These genes in the selected modules were further selected for hub gene analysis.

### 2.7. Identification of Mitochondria-Related Hub Genes

A total of 1136 mitochondria-related genes were obtained from MITOCARTA 3.0 online database [18]. These genes were subsequently intersected with DEGs, and the genes from the key modules were identified through WGCNA. This intersection process was visualized using a Venn diagram, generated with the “ggvenn” package in R (version 0.1.10). The overlapping genes identified through this procedure were designated as immune-related mitochondrial genes and were retained for further machine learning-based selection.

### 2.8. Gene Selection via Multiple Machine Learning Algorithms

To evaluate the diagnostic performance of immune-related mitochondria genes, explainable machine learning algorithms were employed with the help of the “caret” package (version 7.0–1). The four commonly used explainable machine learning models selected for their capacity to support feature importance ranking include random forest (RF), support vector machine (SVM), the generalized linear model (GLM), and extreme gradient boosting (XGBoost), as described in the prior literature [19]. To ensure robust model performance and prevent overfitting, all machine learning models were constructed using a comprehensive hyperparameter tuning process. This was implemented through 5-fold cross-validation, where the training dataset was partitioned into five equal-sized subsets. For each unique combination of hyperparameters, the model was trained on four folds and validated on the remaining fold, with this process repeated five times such that each fold served as the validation set once. The hyperparameter combination that yielded the highest average performance across all five validation folds was selected to build the final model. The diagnostic performance of each machine learning model was evaluated using the receiver operating characteristic curve (ROC). The weight of each gene in the machine learning models was reflected with feature importance, which was calculated by the cooperation of the functions “explainer” and “variable_importance” from the “DALEX” package (version 2.4.3), which was established based on permutation importance analysis. Additionally, the contribution of individual features to model predictions was interpreted using SHAP, with results visualized via the “Shapviz” package (version 0.10.3). The top 20 genes with the highest feature importance in each machine learning model were extracted and intersected to identify common significant hub immune-related mitochondria genes.

### 2.9. PPI Network Construction

The identified hub immune-related mitochondria genes were uploaded to the online STRING database (https://cn.string-db.org/, accessed on 7 September 2025) to analyze the relationship between proteins. The setting parameters were set to the default.

### 2.10. Gene Validation

We validated the gene expression via the external dataset and experimental RT-qPCR. The expression patterns of the identified hub immune-related mitochondrial genes were initially validated using the external dataset, GSE16561. Genes demonstrating consistent expression patterns across both the discovery and validation datasets were selected for further experimental verification.

Gene expression level was experimentally validated with RT-qPCR. Venous blood was collected from 22 stroke patients and 22 age-matched control participants from the First People’s Hospital of Changde City. The inclusion criteria of IS patients include: (1) patients who were diagnosed with IS through Diffusion Weighted Imaging (DWI) sequencing on magnetic resonance imaging (MRI); (2) the diagnosis of IS was made within 24 h of symptom onset. The demographic data was also collected. This validation step was approved by the Ethics Committee of our hospital (2025-069-01) and complied with the Declaration of Helsinki. The samples were centrifuged at 1500× *g* for 10 min to remove the upper plasma layer, and the white blood cell (WBC) layer was collected. Residual red blood cells were lysed using a red blood cell lysis buffer, followed by another centrifugation to collect the WBC pellet. The pellet was lysed thoroughly in 1 mL of TRIzol (Invitrogen (Thermo Fisher Scientific), Carlsbad, CA, USA, #15596018CN). Subsequently, 0.2 mL of chloroform was added, the mixture was vigorously shaken for 15 s, and then left at room temperature for 5 min. After centrifugation at 12,000× *g* for 15 min, RNA was partitioned into the upper aqueous phase. RNA was then precipitated using isopropanol or ethanol and collected by centrifugation. The RNA pellet was washed with 75% ethanol to remove impurities and dissolved in RNase-free water. The RNA concentration was determined using spectrophotometry. Reverse transcription was performed using the HiScript Q RT SuperMix for qPCR (+gDNA wiper) (Vazyme Biotech Co., Ltd., Nanjing, China, #R123–01) to synthesize cDNA from 1 μg of RNA. RT-qPCR was conducted using ChamQ Universal SYBR qPCR Master Mix (Vazyme Biotech Co., Ltd., Nanjing, China, #Q711–02) following the manufacturer’s protocol. The relative gene expression levels were normalized to β-actin mRNA expression and analyzed using the 2^−ΔΔCt^ method. The primers used in this study are shown in the Supplementary Table S1.

### 2.11. MCAO Model

A total of 18 male *C57BL/6* mice (weighing 22–25 g) were used to establish the MCAO model. Mice were randomly assigned to three groups—sham-operated group (SHAM, n = 6), ischemia/reperfusion for 1 day group (IR-1d, n = 6), and ischemia/reperfusion for 3 days group (IR-3d, n = 6)—to evaluate time-dependent changes following cerebral ischemia–reperfusion injury. The mice were obtained from a certified animal facility and housed under specific pathogen-free (SPF) conditions, with a controlled temperature of 22 ± 2 °C, relative humidity of 50–60%, and a 12 h light/dark cycle. Standard chow and water were provided ad libitum.

After shaving and disinfecting the neck, a midline incision was made to expose the surgical field. The left common carotid artery (CCA), external carotid artery (ECA), and internal carotid artery (ICA) were carefully isolated after identifying and preserving the surrounding nerves. The ECA was ligated proximally, and the CCA was temporarily clamped. A small incision was made at the bifurcation of the CCA, and a silicon-coated nylon monofilament (Beijing Cinontech Co., Ltd., Beijing, China, #1620A2) was gently advanced through the ICA to induce transient MCAO. The filament was secured by ligating the CCA, with its tail left external to the closed incision. Throughout the procedure, mice were maintained on a heating pad under a warming lamp. After 1 h of occlusion, the filament was withdrawn under 1.5% isoflurane anesthesia to allow for reperfusion, thus establishing the IR model. Meanwhile, six SHAM mice underwent the same surgical exposure and vessel isolation without insertion of the filament.

All animal experiments were performed under sterile conditions, approved by the institutional ethics committee, and conducted in accordance with the ARRIVE guidelines. Every effort was made to minimize animal suffering and to reduce the number of animals used.

### 2.12. GSEA

The GSEA enrichment algorithms consider the whole-genome expression to explore significant enrichment pathways. In this study, we performed GSEA on each RT-qPCR validated hub gene using the “GSEA” function, visualized via the “gseaplot2” function in the “clusterProfiler” package. For each validated gene, we re-grouped each sample into the high-expression group and the low-expression group based on the highest 25% or the lowest 25% expression level. The differential expression analysis algorithm was employed for the new group, and logFC level results were used to perform GSEA.

### 2.13. Gene Set Variation Analysis (GSVA)

We re-grouped each sample into the high- or low-expression group for each validated gene for GSEA. In GSVA, the functional class score of each enrichment pathway was calculated based on the whole-gene expression level for each sample using the “GSVA” package (version 2.0.1), thus resulting in a new score matrix. Differential analysis of the score matrix was then conducted to identify significant enrichment pathways.

### 2.14. Single-Cell Sequencing Analysis

We downloaded single-cell sequencing data from the GSE174574 dataset, which includes mouse brain samples from three ischemic models that underwent MCAO surgery and three control models that underwent SHAM surgery. The major stages of this single-cell sequencing analysis include quality control, cell cycle control, cell clustering, cell cluster annotation, gene marker analysis, and cell communication analysis. Data for the genes and cells was filtered based on the following criteria: 1. The count of unique molecular identifiers (UMIs) (nCounts) ranged from 200 to 5000. 2. The data complexity, measured by the log-transformed count of genes and UMIs (log10GenesPerUMI), was assessed, indicating the number of genes detected per UMI. 3. A minimum of 200 genes were detected in each cell. 4. The proportion of mitochondrial genes was required to be less than 20%. Cells in the double-cell phase were removed using the “scDblFinder” R package (version 1. 16.0). Following quality control, the remaining cells were clustered using uniform manifold approximation and projection (UMAP). Brain tissue cell markers were obtained from the online repository (http://117.50.127.228/CellMarker/, accessed on 14 November 2025) and matched to each cell cluster for annotation. Marker scores for each cluster were calculated using fully automated cell annotation functions (available at https://github.com/IanevskiAleksandr/sc-type/blob/master/LICENSE, accessed on 14 November 2025). The hub genes identified through this process were considered marker genes, and the expression patterns of these genes across cells were visualized. Finally, cell communication analysis was performed using the “CellChat” package (version 1.6.1), which identified the strength of communication between cells and the pathways involved.

### 2.15. Statistical Analysis

Statistical analysis was performed using the R software (version 4.4.2). Normally distributed numeric data was expressed as mean ± standard deviation (SD) and compared using independent *t*-tests between groups. Non-normally distributed numeric data was expressed as the median (25% quatile–75% quantile) and compared using the non-parametric Wilcox’s test. The significance of differences among three or more groups was analyzed by one-way ANOVA and Kruskal–Wallis tests based on data normality. The area under the receiver operator characteristic (AUC) curve was used to evaluate the diagnostic performance of the machine learning model and hub genes. A *p*-value less than 0.05 indicates that the difference is statistically significant.

## 3. Results

### 3.1. Identification of DEGs in IS Patients and Controls

A total of 2498 DEGs were identified in GSE58294, comprising 1369 downregulated and 1129 upregulated DEGs, between 69 IS patients and 23 control participants (Figure 1).

**Figure 2:**
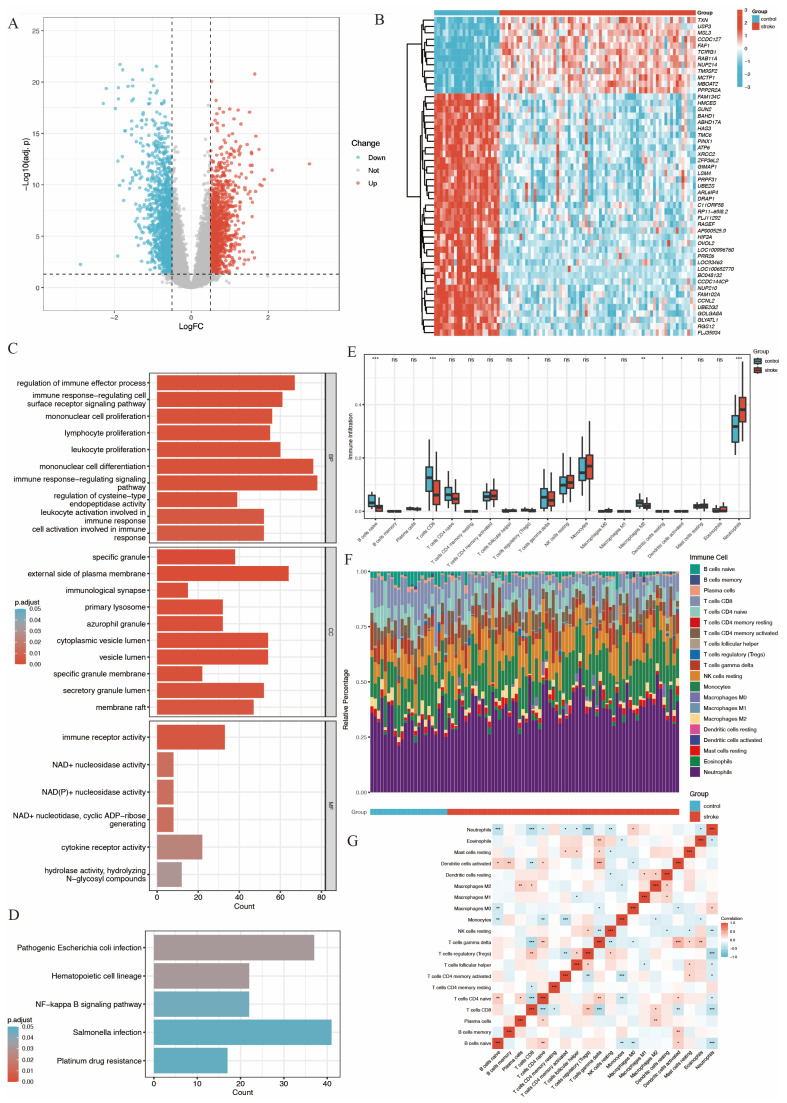
Gene expression analysis of GSE58294 and gene function, immune infiltration analysis. (**A**) Volcano plot of gene expression. (**B**) Heatmap plot of top 50 significant DEGs. (**C**) Bar plot of GO analysis of DEGs, showing biological process (BP), cell components (CC) and molecular function (MF). (**D**) Bar plot of KEGG analysis of DEGs [20,21,22]. (**E**) Boxplots show the differences in immune response between stroke group and control group, with the middle line denoting the median. (**F**) Relative abundance of 22 immune cells between the two groups. (**G**) Pearson correlation heatmap of 22 immune cells (ns. not significant, *p* > 0.05, * *p* < 0.05, ** *p* < 0.01, *** *p* < 0.001).

These DEGs were visualized in a volcano plot (Figure 2A), with upregulated genes colored in red and downregulated genes colored in blue. The top 50 most significant DEGs were visualized using a heatmap plot (Figure 2B).

In addition, we generated a heatmap at various time points within the IS group, as shown in Supplementary Figure S1. This revealed a consistent trend in the expression of the top variate genes within the first 24 h following ischemic stroke onset when compared to the control group.

Gene function analysis of these DEGs was performed using GO and KEGG enrichment analysis. The results (Figure 2C,D) revealed that DEGs were significantly enriched in immune-related functions. Specifically, in the biological process (BP) category, DEGs were associated with processes such as regulation of immune effector activities, immune-response-regulating cell surface receptor signaling, mononuclear cell proliferation, lymphocyte proliferation, leukocyte proliferation, and mononuclear cell differentiation.

An immune infiltration analysis was conducted using the CIBERSORT algorithm to compare IS patients and controls. The analysis indicated a significant decrease in the infiltration of naive B cells, CD8+ T cells, regulatory T cells, M2-type macrophages, resting dendritic cells, and activated dendritic cells in stroke patients. In contrast, neutrophil infiltration was significantly increased, as shown in Figure 2E,F. Furthermore, the increased neutrophil infiltration showed a significant negative correlation with regulatory T cells and CD8+ T cells (Figure 2G).

### 3.2. WGCNA

**Figure 3:**
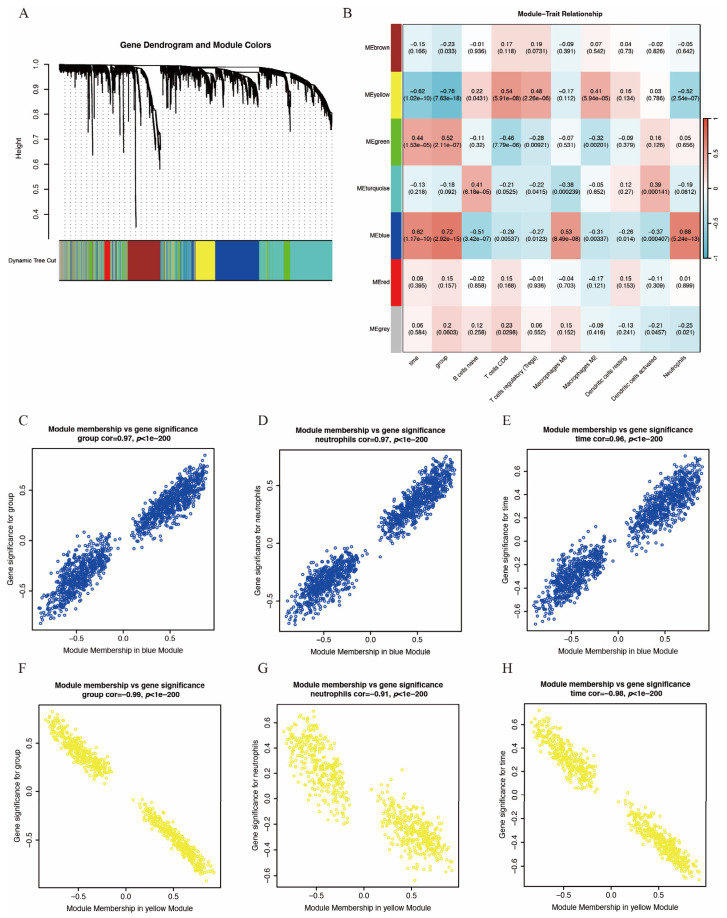
WGCNA of GSE58294. (**A**) Hierarchical clustering dendrogram of co-expression modules, dividing genes into different color segments and representing different modules. (**B**) Heatmap depicting the correlations between modules with several clinical traits and immune scores, with each cell containing the correlation value and *p*-value. (**C**–**E**) Correlation between module membership and gene significance of blue module. (**F**–**H**) Correlation between module membership and gene significance of yellow module. For (**C**–**H**), each point represents a gene, with gene significance indicating the association between the gene and clinical traits and module membership indicating the association between the gene and the module.

To identify key gene modules associated with IS and immune infiltration, we employed the WGCNA algorithm to construct co-expression networks and identify gene modules for the gene expression matrices of both the IS and control groups. Figure 3A,B display the segmentation of six gene modules and their correlation to clinical traits and immune infiltration. The blue module showed the strongest positive correlation with IS and neutrophil infiltration, while the yellow module exhibited the strongest negative correlation. Consequently, these two modules were selected as key modules. Figure 3C–E and Figure 3F–H depict the significant correlation between module membership and gene significance within the blue and yellow modules, respectively. Each point represents a gene, with gene significance indicating the association between the gene and clinical traits and module membership indicating the association between the gene and the module.

### 3.3. Identification of Hub Immune-Related Mitochondrial Genes

**Figure 4:**
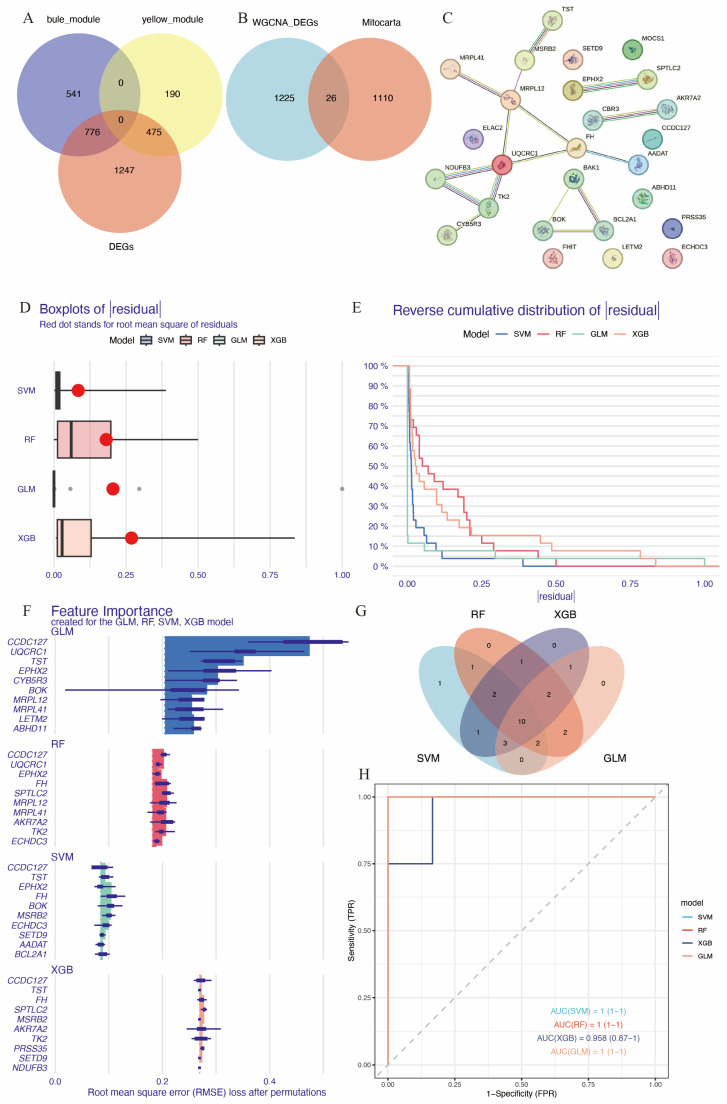
Identification of hub immune-related mitochondrial genes. (**A**) Venn diagram of genes in the blue and yellow modules of WGCNA and DEGs. (**B**) Venn diagram showing genes common in DEGs and key WGCNA modules intersected with mitochondrial genes from Mitocarta database. (**C**) PPI of the 26 immune-related mitochondrial genes. (**D**) Cumulative residual distribution for the four machine learning models trained by training set of GSE58294. (**E**) Boxplot displaying the residuals for each machine learning model, with red dots indicating root mean square errors (RMSEs). (**F**) The top 10 essential gene features of each machine learning model. (**G**) Venn diagram showing the intersection of top 20 essential genes in the four machine learning models, resulting in 10 hub genes. (**H**) ROC analysis of the 10 hub genes based on the testing set of GSE58294.

A total of 1136 mitochondrial-related genes were obtained from the MITOCARTA 3.0 online platform. Overlapping genes between DEGs, the blue and yellow modules from WGCNA, and mitochondrial-related genes were identified using Venn diagrams (Figure 4A,B), resulting in a set of 26 immune-related mitochondrial genes. The protein–protein interaction network for these genes is shown in Figure 4C. To identify hub genes with high diagnostic value, four well-developed machine learning models were employed: random forest (RF), support vector machine (SVM), the generalized linear model (GLM), and extreme gradient boosting (XGB). Samples in GSE58294 were divided into training and testing subsets in a 3:1 ratio. The training subset was used to train the machine learning models, and models SVM, GLM, and XGB exhibited relatively low residuals (Figure 4D,E). The top 10 essential feature variables for each model were ranked based on the root mean square error (RMSE) (Figure 4F). To evaluate the discriminative performance of the models, ROC curve analysis was performed. The area under the curve (AUC) values for the four machine learning models were high and comparable: GLM, AUC = 1; SVM, AUC = 1; RF, AUC = 1; XGBoost, AUC = 0.958 (0.87–1) (Figure 4H).

The contribution of each gene to the model predictions was visualized using SHAP (SHapley Additive exPlanations) analysis (Supplementary Figure S2). The top 20 essential feature variables from each model were selected for Venn diagram intersection analysis (Figure 4G), leading to the identification of 10 common immune-related mitochondrial genes: *FH*, *MSRB2*, *ECHDC3*, *CCDC127*, *SPTLC2*, *MOCS1*, *EPHX2*, *TK2*, *CYB5R3*, and *MRPL41.*

### 3.4. Validation of Hub Immune-Related Mitochondrial Genes

**Figure 5:**
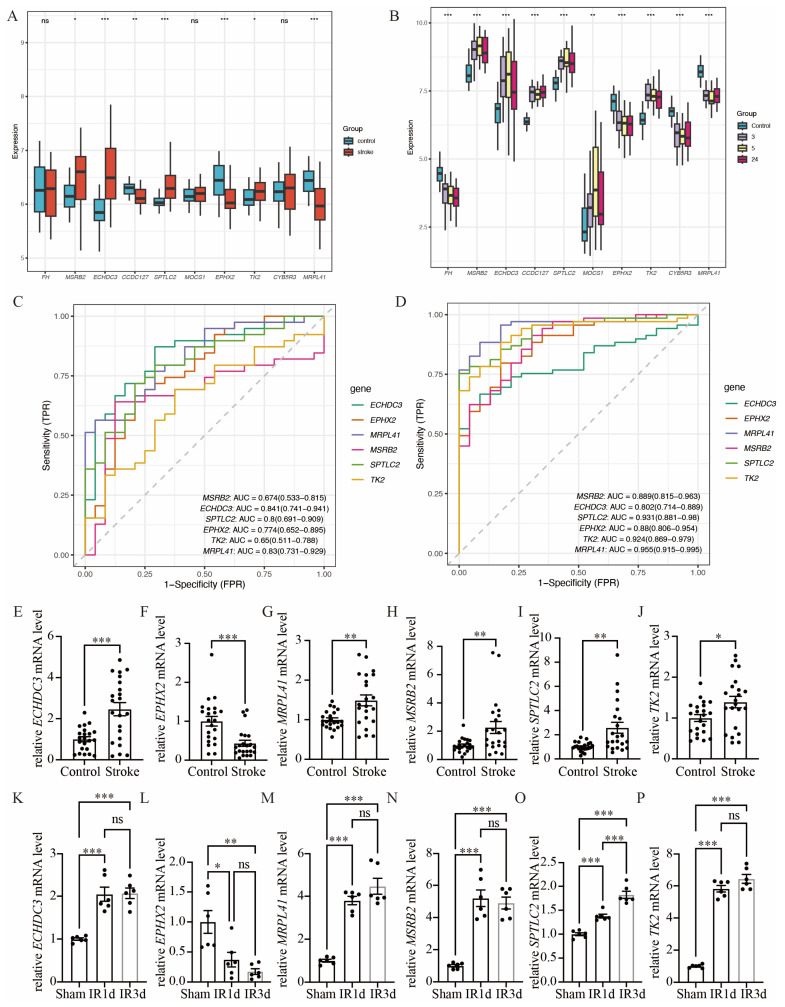
Validation of hub immune-related mitochondrial genes. (**A**) Boxplots show the expression of 10 hub immune-related mitochondrial genes between stroke group and control group in GSE16561. (**B**) Boxplots show the expression of 10 hub immune-related mitochondrial genes between stroke group and control group in GSE16561, in which the gene expression of stroke group was separated into three time points: 3 h, 5 h and 24 h after stroke onset. (**C**) ROC analysis of the six hub genes with same pattern of expression in both GSE58294 and GSE16561 based on GSE16561. (**D**) ROC analysis of the six hub genes with same pattern of expression in both GSE58294 and GSE16561 based on GSE58294. (**E**–**J**) RT-qPCR validation of the expression levels of the six hub genes, n = 22. (**K**–**P**) Compared with the SHAM group, the mRNA expression levels of the six hub genes in the brain tissue of mice subjected to ischemia–reperfusion (IR) were determined by RT-qPCR, n = 6 (ns. not significant, * *p* < 0.05, ** *p* <0.01, *** *p*< 0.001). The middle line in (**A**,**B**) and (**E**–**P**) denotes the median and each dot represents the value of an individual sample.

We used GSE16561 as an external validation dataset to analyze the gene expression levels of the common hub immune-related mitochondrial genes (Figure 5A). The expression pattern in GSE16561 was compared to that in GSE58294 (Figure 5B), and genes that exhibited the same direction of differential expression across both datasets were selected for further RT-qPCR analysis. This process identified six candidate genes: *ECHDC3*, *EPHX2*, *MRPL41*, *MSRB2*, *SPTLC2*, and *TK2*. We further validated these six genes using the RT-qPCR method on blood samples collected from acute ischemic stroke patients and control participants (Figure 5E–J). The baseline characteristics of the patients in the IS and control groups showed no significant differences (Supplementary Table S2). All six genes exhibited significant expression differences between the stroke group and the control group. However, the expression pattern of MRPL41 was inconsistent with the findings from both GSE58294 and GSE16561. In addition, we validated these hub genes in the mouse middle cerebral artery occlusion (MCAO) and SHAM model. Total RNA was extracted from the brain tissues of MCAO and SHAM mice, and gene expression was detected by RT-qPCR. The expression patterns of the five genes (*ECHDC3*, *EPHX2*, *MSRB2*, *SPTLC2*, and *TK2*) in mouse brain tissues were consistent with those observed in human peripheral blood samples (Figure 5K–P), further supporting their potential as conserved immune-related mitochondrial markers in ischemic stroke. Specifically, following ischemia–reperfusion (IR) injury, the expression of *ECHDC3*, *MSRB2*, *SPTLC2*, and *TK2* showed a significant upregulation trend at both IR1d and IR3d time points, with *SPTLC2* exhibiting the most pronounced increase at IR3d. In contrast, the expression of *EPHX2* was markedly downregulated after IR, displaying an opposite expression pattern compared to the other genes. These results suggest that the expression changes in these genes after cerebral ischemia are time-dependent and direction-specific and may collectively participate in mitochondrial-related immune-regulatory processes. Thus, *ECHDC3*, *EPHX2*, *MSRB2*, *SPTLC2*, and *TK2* were considered the hub immune-related mitochondrial genes in this study.

The diagnostic performance of these six genes was evaluated in both GSE16561 (Figure 5C) and GSE58294 (Figure 5D).

In the GSE58294 dataset, the diagnostic performance of these genes was high, with the following AUC values: *ECHDC3* AUC = 0.802 (0.714–0.889), *EPHX2* AUC = 0.880 (0.806–0.954), *MRPL41* AUC = 0.955 (0.915–0.995), *MSRB2* AUC = 0.889 (0.815–0.963), *SPTLC2* AUC = 0.931 (0.881–0.980), and *TK2* AUC = 0.924 (0.869–0.979). In the GSE16561 dataset, four of these six genes exhibited diagnostic performance above 0.7, with the following AUC values: *ECHDC3* AUC = 0.841 (0.741–0.941), *EPHX2* AUC = 0.774 (0.652–0.895), *MRPL41* AUC = 0.830 (0.731–0.929), and *SPTLC2* AUC = 0.800 (0.691–0.909).

**Figure 6:**
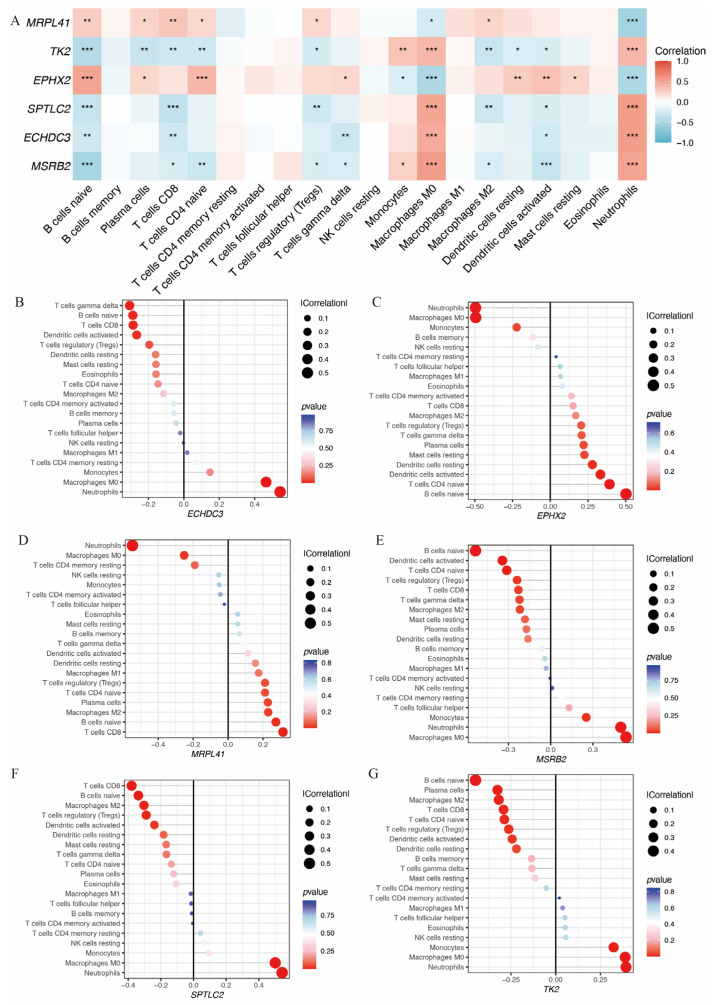
Immune infiltration analysis of the hub immune-related mitochondrial genes. (**A**) Heatmap shows the correlation between hub gene expression and immune infiltration cells. (**B**–**G**) Lollipop plots depict the correlation between expression levels of hub genes and immune infiltration cells (* *p* < 0.05, ** *p* < 0.01, *** *p*< 0.001).

Additionally, the relationship between these six genes and immune infiltration was assessed (Figure 6A). All six genes were significantly associated with neutrophil infiltration. *EPHX2* and *MRPL41* showed a negative correlation, while *TK2*, *SPTLC2*, *ECHDC3*, and *MSRB2* showed positive correlations. Figure 6B–G illustrate the associations of each gene with 22 distinct immune cell types.

**Figure 7:**
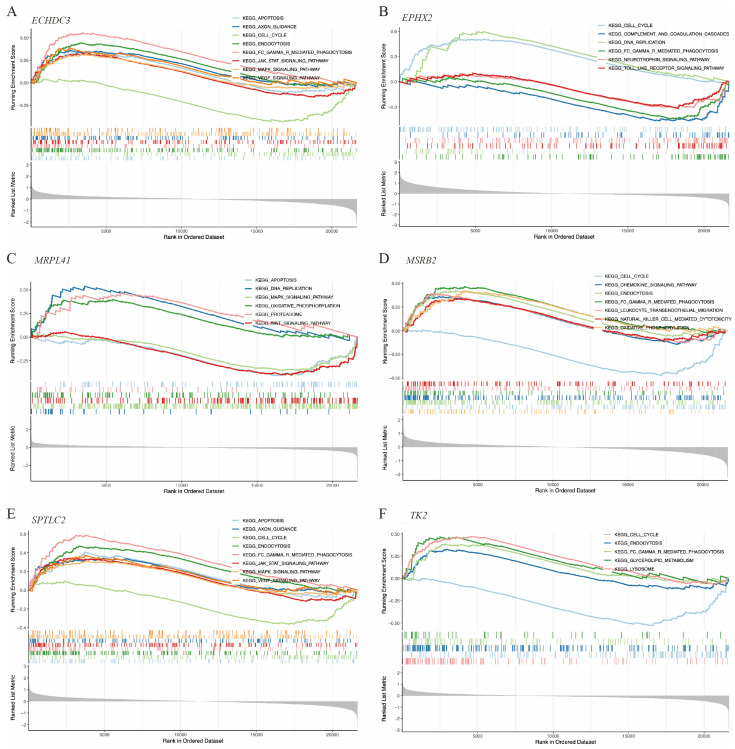
GSEA of the hub immune-related mitochondrial genes. (**A**–**F**) show pathways enriched by GSEA associated with *ECHDC3*, *EPHX2*, *MRPL41*, *MSRB2*, *SPTLC2*, and *TK2*, respectively.

Gene function enrichment was conducted using GSEA and GSVA algorithms. The GSEA results indicated that *ECHDC3*, *MRPL41*, and *SPTLC2* were involved in the cell apoptosis process. *ECHDC3* and *SPTLC2* were associated with the JAK-STAT, MAPK, and VEGF signaling pathways, while *MRPL41* was associated with the WNT and MAPK signaling pathways. All of these genes, except *MRPL41*, were associated with the cell cycle. *ECHDC3*, *MSRB2*, *TK2*, and *SPTLC2* were linked to the endocytosis process (Figure 7A–F).

**Figure 8:**
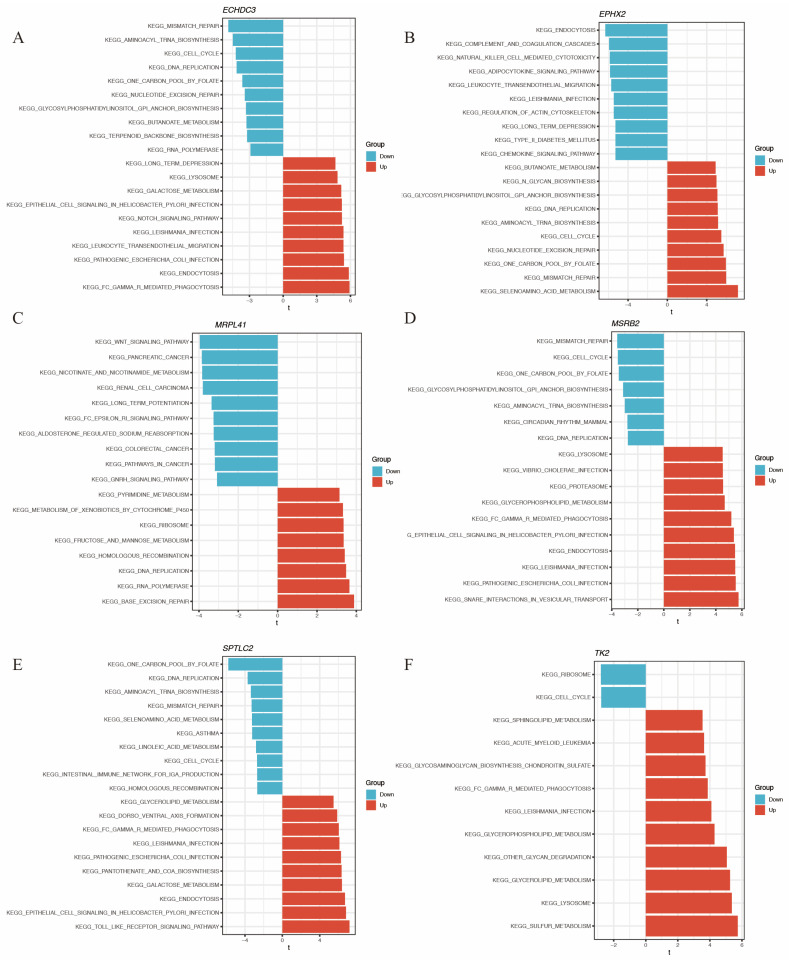
GSVA of the hub immune-related mitochondrial genes. (**A**–**F**) show pathways enriched by GSVA associated with *ECHDC3*, *EPHX2*, *MRPL41*, *MSRB2*, *SPTLC2*, and *TK2*, respectively.

The direction of association between these genes and functional pathways is shown in Figure 8A–F.

### 3.5. Single-Cell Sequencing Analysis

**Figure 9:**
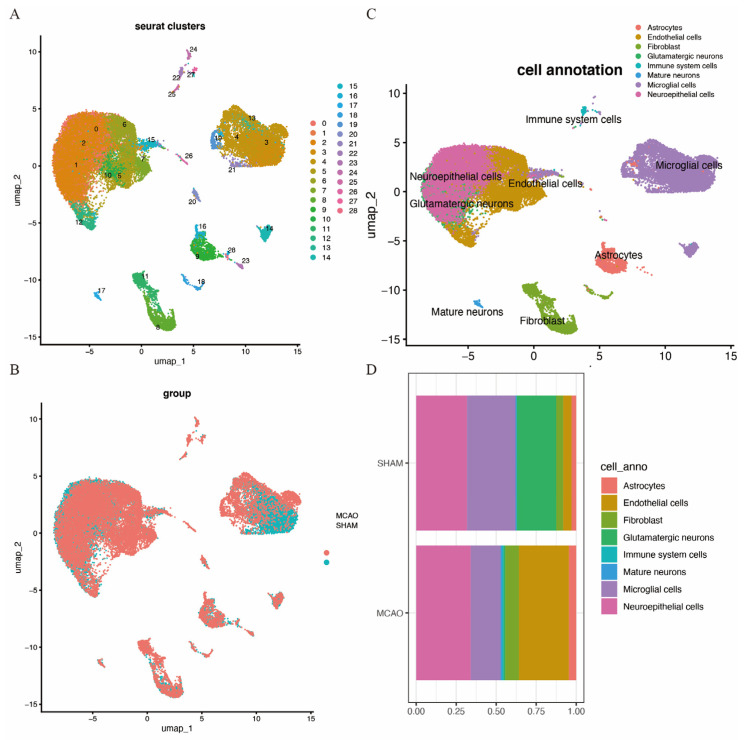
Cell clusters of single-cell sequencing. (**A**) cUMAP map with 28 distinct clusters. (**B**) UMAP map with eight cell types annotated to cell clusters. (**C**) UMAP cluster by group. (**D**) Histogram of cell-type content for two groups.

A quality control procedure was performed on the GSE174574 dataset. As shown in Supplementary Figure S3, cells with low UMIs, low RNA features and a high ratio of mitochondrial expressed genes were removed. This process reduced the total cell count from 58,523 to 36,667. Following quality control, UMAP clustering was performed, resulting in 28 clusters (Figure 9A). The clustering of cells from MCAO and SHAM brain tissues is depicted in Figure 9B. Further cell annotation identified eight distinct cell types, including astrocytes, endothelial cells, fibroblasts, glutamatergic neurons, immune cells, mature neurons, microglia, and neuroepithelial cells, as shown in Figure 9C. Figure 9D highlights the distribution of these cell types in the two mouse models, revealing an increase in endothelial cells, fibroblasts, neuroepithelial cells, and immune cells in the MCAO brain, with a decrease in microglial cells and glutamatergic neurons.

**Figure 10:**
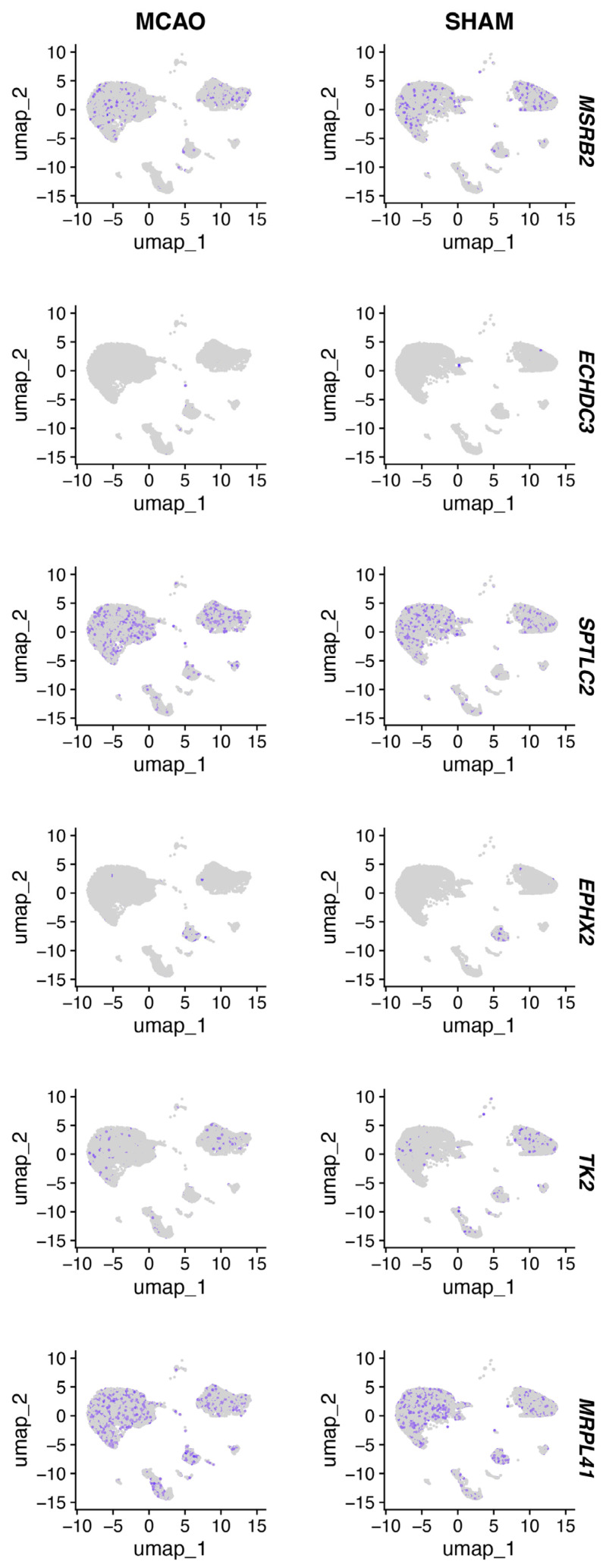
UMAP plot of hub immune-related mitochondrial genes.

Figure 10 shows *ECHDC3*, *EPHX2*, *MSRB2*, *SPTLC2*, and *TK2* expression patterns in the mouse brain tissue of the MCAO and SHAM models.

**Figure 11:**
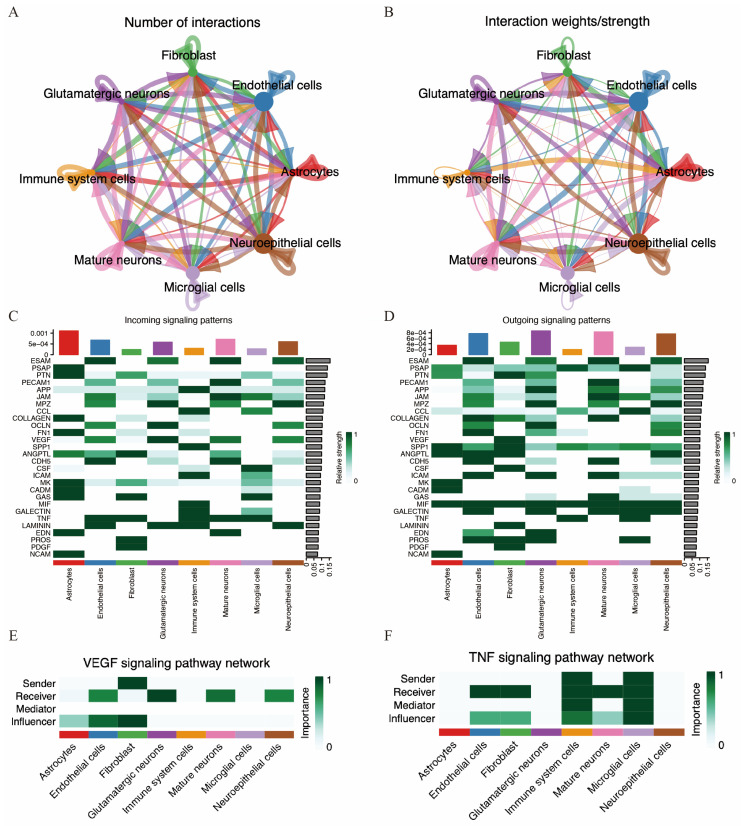
Cell communication in MCAO mouse brain. (**A**,**B**) Number of cell–cell interactions and total interaction strength. (**C**,**D**) Heatmaps displaying the intensity of incoming and outgoing interactions among different cell subpopulations. (**E**,**F**) Strength of VEGF and TNF signaling pathway among different cell types.

To better understand the microenvironment and cell-to-cell interactions in acute ischemic stroke, we performed cell communication analysis using the “CellChat” package on the single-cell RNA sequencing data from the MCAO group. The interactions between various cell types are illustrated in Figure 11A,B.

We further investigated potential efferent and afferent signals, as well as specific ligand–receptor pairs among these cell types. A total of 28 signal pathways between cell types were identified. Immune cells were identified as potential receptors for several pathways, including APP, CCL, SPP1, ICAM, MIF, GALECTIN, TNF, and LAMININ. Conversely, immune cells also acted as signal providers for pathways such as TNF, MIF, SPP1, CCL, and PSAP (Figure 11C,D). Notably, the VEGF pathway was potentially sent by fibroblasts and received by endothelial cells, glutamatergic neurons, mature neurons, and neuroepithelial cells. Additionally, the TNF pathway was potentially sent by both immune cells and microglia and received by endothelial cells, fibroblasts, immune cells, mature neurons, and microglia.

## 4. Discussion

Mitochondria, as the central organelles for energy metabolism, play a crucial role in the pathological process of IS. Disruption of cerebral blood flow leads to a cessation of oxygen and nutrient supply, impairing mitochondrial oxidative phosphorylation and resulting in insufficient ATP synthesis, which severely affects neuronal excitability and survival [23]. Additionally, cerebral ischemia is often accompanied by excessive production of reactive oxygen species (ROS), and the overaccumulation of ROS and calcium ions disrupts the stability of both the inner and outer mitochondrial membranes [24,25]. This activation of the mitochondrial permeability transition pore (MPTP) leads to mitochondrial swelling and membrane rupture, releasing large amounts of cytochrome c, which triggers the apoptotic cascade and ultimately induces cell death [26]. Therefore, targeting mitochondria-related genes to regulate mitochondrial function may offer a new therapeutic direction for IS.

In this study, we utilized GSE16561 as the external validation dataset to analyze the gene expression levels of common hub immune-related mitochondrial genes. By comparing expression patterns between GSE16561 and GSE58294, we selected genes showing consistent differential expression and subsequently conducted RT-qPCR analysis. This process identified six mitochondrial-related differentially expressed genes associated with IS pathogenesis: *ECHDC3*, *EPHX2*, *MRPL41*, *MSRB2*, *SPTLC2*, and *TK2*. Functional enrichment analysis revealed that these genes are predominantly involved in apoptosis, oxidative stress response, and lipid metabolism. Notably, all six genes demonstrated significant associations with neutrophil infiltration, suggesting their potential involvement in the immunometabolic crosstalk underlying IS pathogenesis.

Our single-cell analysis of the GSE174574 dataset revealed cell-type-specific expression patterns of these six mitochondrial hub genes in a mouse MCAO model. *MRPL41* and *TK2* were predominantly expressed in neurons, associating with p53-mediated apoptosis and mitochondrial DNA maintenance, respectively. *MSRB2* and *SPTLC2* localized to microglia, linking mitochondrial repair and ceramide synthesis to neuroinflammation. *EPHX2* was enriched in pericytes, supporting its vasoprotective role [27], while *ECHDC3* was expressed in endothelial cells, suggesting a role in metabolic adaptation. CellChat analysis identified disrupted VEGF-MAPK signaling between endothelial cells and pericytes, alongside microglia-mediated neutrophil recruitment via S100a8/a9-Tlr4 interactions. These findings complement bulk RNA-seq data by resolving cell-type-specific mechanisms and highlighting non-cell-autonomous crosstalk in IS.

*ECHDC3*, as an important protein in mitochondria, functions as 3-hydroxyacyl-CoA dehydrogenase and enoyl-CoA hydratase [28]. These enzymes are involved in the β-oxidation pathway, fatty acid metabolism, and the function of the mitochondrial respiratory chain [29,30,31]. Duarte MK et al. [32] found a correlation between the upregulation of *ECHDC3* and serum unsaturated fatty acids with the severity of acute coronary syndrome. Although a direct link to IS has not been established, abnormalities in fatty acid metabolism are intricately linked to IS pathogenesis. Our bioinformatics analysis revealed significant upregulation of *ECHDC3* following cerebral ischemia, suggesting its involvement in disease onset and progression. This upregulation may reflect a compensatory attempt to restore energy balance via enhanced lipid oxidation. Its enrichment in MAPK and VEGF pathways implies potential roles in inflammation and angiogenesis. Given its positive correlation with neutrophil infiltration, *ECHDC3* may serve as a metabolic modulator linking mitochondrial function to immune activation.

The *EPHX2* gene encodes an enzyme that is a member of the cytochrome P450 2E1 (CYP2E1) family. This enzyme is involved in redox reactions and lipid metabolism, and its function is closely related to cellular inflammatory responses and oxidative stress. Variations in the *EPHX2* gene or changes in its expression levels may be associated with the risk of stroke. Fornage et al. [33] analyzed 12 single-nucleotide polymorphisms (SNPs) of *EPHX2* in African American and Caucasian populations, suggesting that *EPHX2* may be either a protective or a risk factor for ischemic stroke. Fava et al. [34] found that the functional K55R polymorphism of the *EPHX2* gene increased the risk of hypertension and ischemic stroke in male homozygotes. Shao et al. [35] studied the relationship between genetic polymorphisms associated with the cytochrome P450 (CYP) metabolic pathway and stroke susceptibility in acute ischemic stroke patients with carotid plaque in southeast China. They found that the *EPHX2* G860A polymorphism may reduce susceptibility to stroke. Cerebral infarction is typically associated with the generation of a large amount of reactive oxygen species, and *EPHX2* helps to clear toxic substances from the body by hydrolyzing epoxides, thereby maintaining vascular health and potentially influencing the prognosis of stroke. In our study, *EPHX2* was significantly downregulated in stroke samples. This reduction may result in the accumulation of toxic lipid epoxides and impaired vascular homeostasis. Importantly, the negative association between *EPHX2* and neutrophil infiltration supports the hypothesis that sEH may exert protective effects by maintaining endothelial integrity and limiting immune cell infiltration. Thus, targeting *EPHX2* could represent a therapeutic strategy to modulate post-ischemic inflammation.

*MRPL41*, also known as *BMRP*, is a binding partner of Bcl-2. By binding to and blocking the anti-apoptotic activity of Bcl-2, it is closely related to cell apoptosis [36]. In tumor cells, MRPL41 enhances the stability of p53 while promoting its translocation to the mitochondria, leading to cell apoptosis [37]. However, the role of *MRPL41* in ischemic stroke remains unclear. Guo et al. [38] found that the mRNA and protein levels of *MRPL41* were upregulated after ischemia–reperfusion. *MRPL41* exacerbated the cerebral ischemia phenotype by activating the p53 pathway. Consistently, we observed an upregulation of *MRPL41* expression in a mouse model of cerebral ischemia, supporting its role in promoting apoptosis during the early phase of ischemic injury. However, our bioinformatics analysis suggested that *MRPL41* expression is downregulated after cerebral infarction, whereas RT-qPCR validation in peripheral blood from stroke patients showed upregulated expression. This discrepancy may be attributed to patient heterogeneity, including stroke subtype, severity, and comorbidities, as well as dynamic temporal changes in *MRPL41* expression, with differences in sampling time points causing variable expression levels across studies. Furthermore, as a target regulated by the p53 pathway, *MRPL41* expression may be subject to post-transcriptional regulation, resulting in discordance between mRNA and protein levels. Mitochondrial dysfunction and altered protein synthesis capacity may also further modulate *MRPL41* expression. Taken together, *MRPL41* exhibits complex dynamic regulation in ischemic stroke and plays a critical role in apoptosis, mitochondrial function maintenance, and oxidative stress, potentially acting as a molecular switch balancing neuronal survival and death. Future studies integrating clinical subgroup analyses and multi-time point, multi-sample transcriptomic and proteomic data are warranted to elucidate the precise mechanisms and clinical relevance of *MRPL41* in ischemic stroke.

*MSRB2* is an enzyme that exerts antioxidant effects by repairing oxidative damage to protein residues caused by oxidation [39]. Additionally, it may play an important role in mitochondrial clearance. Lee et al. [40] proposed that after mitochondrial damage, *MSRB2* is released from the mitochondrial matrix, where it promotes mitophagy by reducing the oxidation of methionine (MetO) in Parkin. It also mediates Parkin ubiquitination while interacting with LC3, facilitating the process of mitochondrial autophagy. The upregulation of *MSRB2* after stroke observed in our study may represent an adaptive response to counteract oxidative damage and remove dysfunctional mitochondria. Its positive correlation with neutrophil infiltration raises the possibility that *MSRB2* also participates in modulating immune responses, possibly by reducing mitochondrial-derived danger signals and limiting secondary inflammatory injury.

*SPTLC2* is one of the key enzymes involved in the synthesis of sphingolipids, particularly in the process of acylating long-chain sphingoid bases. Sphingolipids are important lipid molecules primarily found in the cell membranes and myelin sheaths of nerve cells, playing a crucial role in the structure and function of neurons [41,42]. Neuronal damage is associated with disturbances in sphingolipid metabolism, and abnormalities in *SPTLC2* may affect the synthesis of neuro-sphingolipids, thereby impacting the function of the nervous system. Huang et al. [43] proposed that after brain ischemia, the levels of long-chain ceramides are significantly elevated, which induces astrocyte activation. This, in turn, triggers oxidative stress and further disrupts mitochondrial homeostasis by increasing mitochondrial permeability. The increased expression of *SPTLC2* observed in our data may contribute to stroke pathology by facilitating ceramide accumulation and glial activation. Functional annotation indicated *SPTLC2* involvement in the JAK-STAT and MAPK signaling pathways, suggesting that it may also influence cytokine signaling and neuroinflammation. Knockdown of *SPTLC2* reduces ceramide production, leading to diminished astrocyte activation and attenuating brain ischemia-induced damage.

*TK2* is a deoxynucleoside kinase responsible for catalyzing the conversion of thymidine to thymidine monophosphate [44]. It is constitutively expressed in the mitochondria and plays a crucial role in mitochondrial DNA replication and energy metabolism [45]. Mutations in *TK2* are associated with severe mitochondrial DNA depletion syndromes. The development of *TK2* inhibitors may aid in studying the role of *TK2* in mitochondrial homeostasis, as well as its involvement in the mitochondrial toxicity observed in patients undergoing long-term treatment with anticancer and antiviral drugs [46]. Oxidative stress and hypoxic conditions induced by stroke can lead to mitochondrial DNA damage, and *TK2* may be vital for maintaining the integrity of mitochondrial DNA. In this study, *TK2* is considered a potential biomarker for neurodamage following stroke. Its association with cell cycle pathways and positive correlation with immune cell infiltration highlight *TK2* as a potential biomarker for neuronal repair and neuroinflammation in stroke.

Additionally, we found that all six genes were significantly associated with neutrophil infiltration. *EPHX2* and *MRPL41* showed negative correlations, while *TK2*, *SPTLC2*, *ECHDC3*, and *MSRB2* exhibited positive correlations. Functional enrichment analysis indicated that *ECHDC3*, *MRPL41*, and *SPTLC2* are involved in apoptosis. *ECHDC3* and SPTLC2 are associated with JAK-STAT, MAPK, and VEGF signaling, while *MRPL41* is related to WNT and MAPK signaling. All genes except *MRPL41* are involved in the cell cycle. These findings suggest that these genes play crucial roles in cerebral ischemia–reperfusion injury and may serve as potential biomarkers and therapeutic targets for neurological damage following IS.

This study has several limitations. First, the GSE58294 dataset comprises patients with cardioembolic stroke, while the GSE16561 dataset lacks detailed stroke subtype information. Additionally, the 22 clinical validation cases were not classified according to the TOAST criteria. This inconsistency in subtype information may limit the applicability of our findings across different IS etiologies. Future studies should stratify patients by well-defined subtypes to explore differential expression patterns and mechanisms of these key genes. Furthermore, the small sample size for RT-qPCR validation (22 IS cases) may reduce statistical power and limit generalizability. Future research should involve larger, subtype-specific, well-characterized clinical cohorts with longitudinal follow-up, preferably in a multicenter setting, to strengthen the robustness and translational relevance of our findings.

In summary, we identified six immune-related mitochondrial genes via WGCNA, machine learning, and external validation. Five of these genes (*ECHDC3*, *EPHX2*, *MSRB2*, *SPTLC2*, and *TK2*) were experimentally validated by RT-qPCR. Functional enrichment analysis revealed their significant associations with the apoptosis, JAK-STAT, MAPK, and VEGF signaling pathways. This study highlights the potential role of immune-related mitochondrial genes in IS pathogenesis, providing new insights and a theoretical foundation for early diagnosis and targeted therapy of IS.

## Data Availability

The gene datasets used in this study are available on the GEO database. The R codes used in this study are available from the corresponding author upon reasonable request.

## Supplementary Materials

The following supporting information can be downloaded at https://www.mdpi.com/article/10.3390/biomedicines14020375/s1: Table S1 presents the primer sequences employed in RT-qPCR. Table S2 summarizes the demographic data of the participants involved in gene validation. Figure S1 shows the heatmap of the top 50 most variable genes in the GSE16561 dataset with IS group separate into three timepoints. Figure S2 shows the SHAP value for explanation of the four machine learning models. Figure S3 shows the quality control process of GSE174574 for single cell analysis.
