# Supplementary material for: Comprehensive Analysis of Immune-Related Mitochondrial Genes in Ischemic Stroke Through Integrated Bioinformatics and Validation
Source: Biomedicines. 2026 Feb 5;14(2):375. doi: 10.3390/biomedicines14020375 (PMC12938822; doi:10.3390/biomedicines14020375)
Supplement: Supplementary file 1 [file biomedicines-14-00375-s001.zip › Supplementary Figures.pdf]

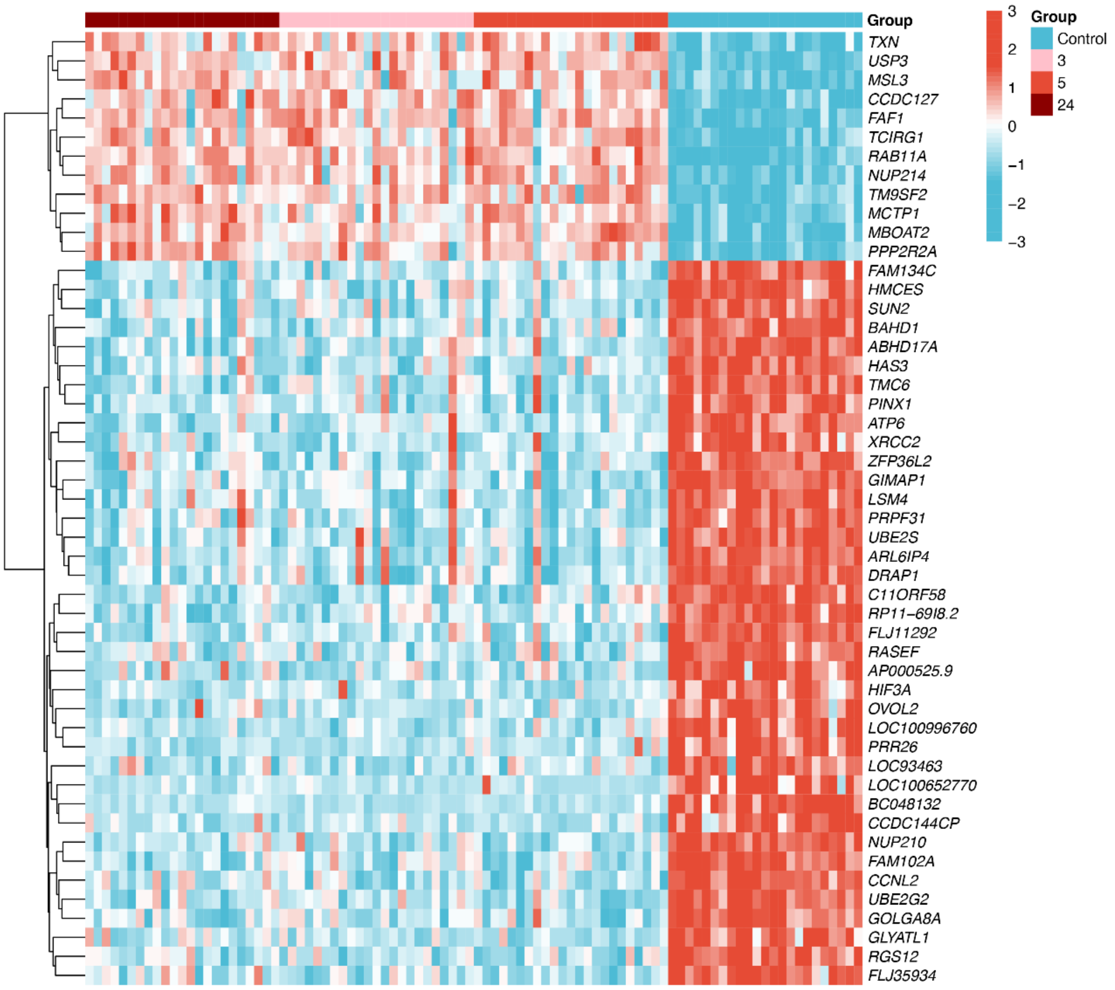

Figure S1 gene expression heatmap with different time points in IS group from GSE16561

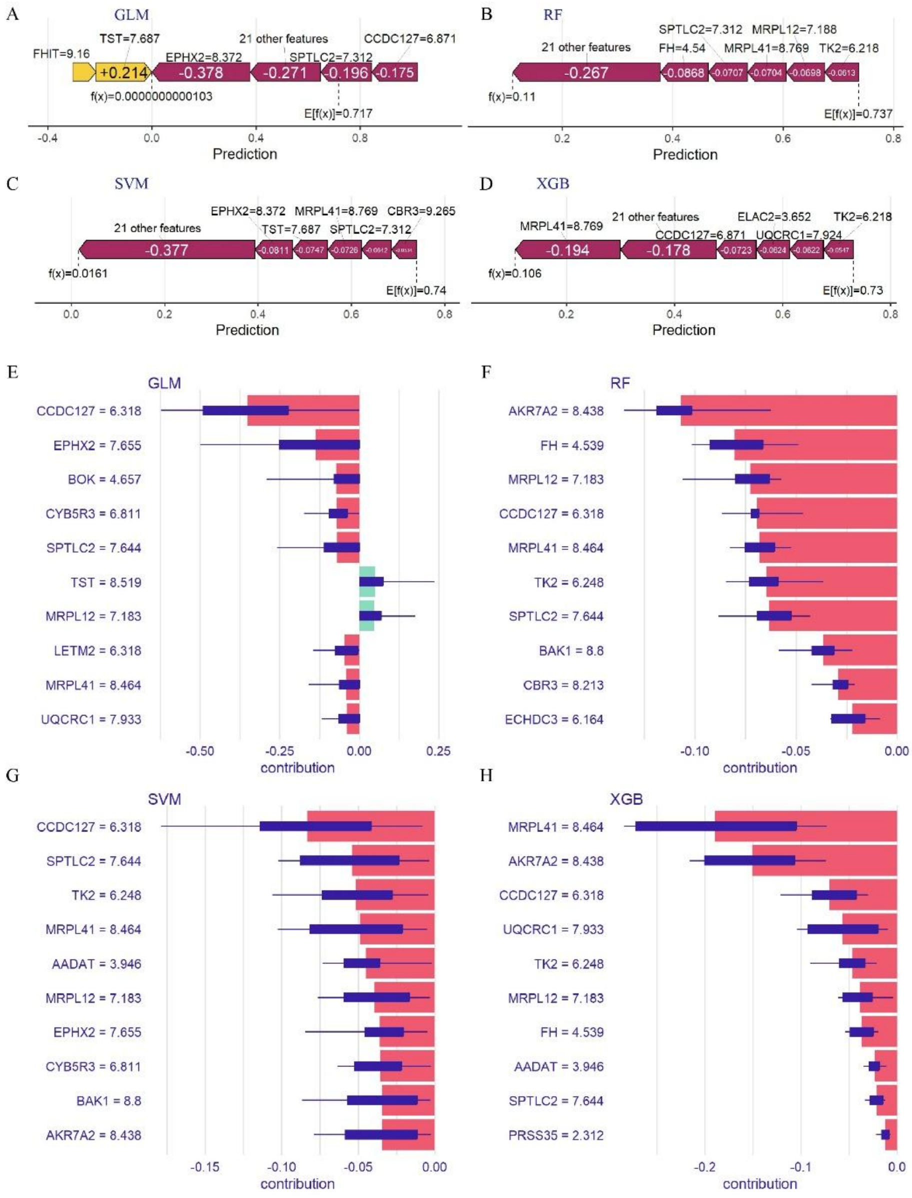

Figure S2 SHAP analysis for explanation of machine learning models

Figure A–D display force plots that visualize the SHAP values of individual genes and their impact on the model's predictions for specific instances. Figure E–H show feature importance plots that utilize the mean absolute SHAP values to evaluate the overall contribution of each gene to the model. Features in these plots are arranged in descending order of importance, with the most influential features at the top.

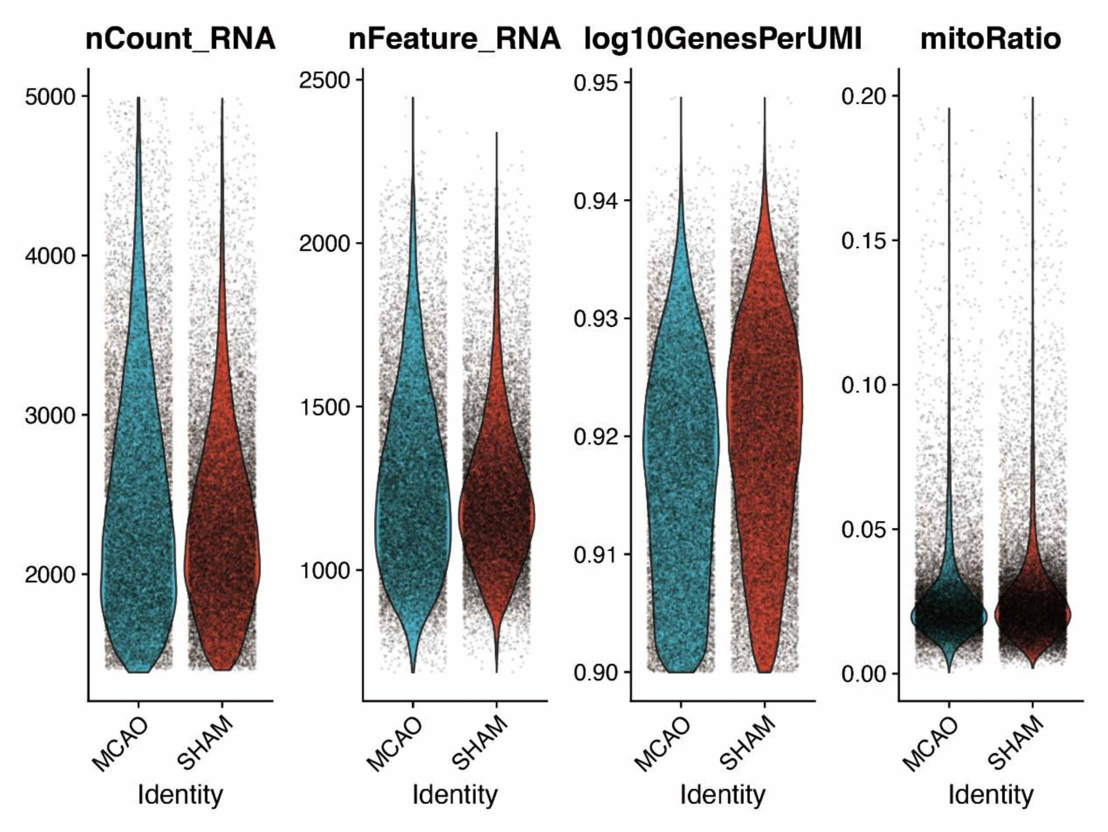

Figure S3 quality control of GSE174574 dataset
