# Supplementary material for: Comprehensive Analysis of Immune-Related Mitochondrial Genes in Ischemic Stroke Through Integrated Bioinformatics and Validation
Source: Biomedicines. 2026 Feb 5;14(2):375. doi: 10.3390/biomedicines14020375 (PMC12938822; doi:10.3390/biomedicines14020375)
Supplement: Supplementary file 1 [file biomedicines-14-00375-s001.zip › Supplementary Tables.pdf]

Supplementary Table S1. Primers of RT-qPCR

Genes	Species	Direction	Sequences
ECHDC3	human	Forward	5′-CTGGACGGCATAAGGAACATC-3′
		Reverse	5′-GACTTTCAGATCGTTGCTGTCA-3′
EPHX2	human	Forward	5′-GTGCTCCGAGACCGCTAAAG-3′
		Reverse	5′-GCTGAAATCGCCTTGTCAAAGAT-3′
MRPL41	human	Forward	5′-GTTCGTCGTCCCGGATCTG-3′
		Reverse	5′-GTAGCTCACGTAGGGCTTGA-3′
MSRB2	human	Forward	5′-CGGAGCAGTTCTACGTCACAA-3′
		Reverse	5′-CGGAGCAGTTCTACGTCACAA-3′
SPTLC2	human	Forward	5′-TGGGTTCCTACAACTATCTTGGA-3′
		Reverse	5′-CATACGCCATAGCAGCTTCTAC-3′
TK2	human	Forward	5′-GGCCTGATGTACCACGATGC-3′
		Reverse	5′-CCTGAGGACGAGTATGCCTG-3′
β-actin	human	Forward	5′-CATGTACGTTGCTATCCAGGC-3′
		Reverse	5′-CTCCTTAATGTCACGCACGAT-3′
ECHDC3	mouse	Forward	5′-CGACAGCAGGACGGAATCAG-3′
		Reverse	5′-GTGAAGAATGTCACTTCGGAGAG-3′
EPHX2	mouse	Forward	5′-CTTGGTGCGTACCAGACGG-3′
		Reverse	5′-TTCTCAGGTAGATTGGCTCCA-3′
MRPL41	mouse	Forward	5′-CTGACTGCCGTGACTCAAGG-3′
		Reverse	5′-CACCCCGACTCTTAGTGAAGG-3′
MSRB2	mouse	Forward	5′-GGGTCTCTTACAAAGTCCAAGC-3′
		Reverse	5′-CACAATGGTACATCCCTGTCTC-3′
SPTLC2	mouse	Forward	5′-GTGAGGAACGGGTACTTGAGG-3′
		Reverse	5′-CAACCAGCATGGGTGTTTCTT-3′
TK2	mouse	Forward	5′-AGCAGTGGTTTGTATTGAGGG-3′
		Reverse	5′-ACATGAGGCTCAGAGGGTTATG-3′
β-actin	mouse	Forward	5′-GGAGATCACAGCTCTGGCT-3′
		Reverse	5′-GTCGATTGTCGTCCTGAGG-3′

Supplementary Table S2. Comparison of Clinical Characteristics Between Ischemic Stroke Patients and Controls

Characteristics	IS (n=22)	Control (n=22)	p
Age (SD)	65.5(10.1)	69.8 (8.0)	0.127
Male (%)	15 (68.2)	14 (63.6)	1.000
Hypertension (%)	19 (86.4)	14 (63.6)	0.164
Cerebral atherosclerosis (%)	19 (86.4)	18 (81.8)	1.000
